# Monocytes carrying GFAP detect glioma, brain metastasis and ischaemic stroke, and predict glioblastoma survival

**DOI:** 10.1093/braincomms/fcaa215

**Published:** 2020-12-26

**Authors:** Wouter B L van den Bossche, Arnaud J P E Vincent, Cristina Teodosio, Jeroen Koets, Aladdin Taha, Anne Kleijn, Sandra de Bruin, Willem A Dik, Daniela Damasceno, Julia Almeida, Diederik W J Dippel, Clemens M F Dirven, Alberto Orfao, Martine L M Lamfers, Jacques J M van Dongen, for the TiMaScan Research Group

**Affiliations:** 1 Department of Neurosurgery, Brain Tumour Center, Erasmus MC, Rotterdam, The Netherlands; 2 Department of Immunology, Leiden University Medical Center, Leiden, Netherlands; 3 Department of Immunology, Erasmus MC, Rotterdam, Netherlands; 4 Department of Neurology, Erasmus MC, Rotterdam, Netherlands; 5 Cytometry Service and Department of Medicine, Cancer Research Center (IBMCC-CSIC/USAL), University of Salamanca, IBSAL and CIBERONC, Salamanca, Spain

**Keywords:** glioma, metastatic CNS tumour, acute ischemic stroke, neuroimmunology, minimal invasive diagnostics

## Abstract

Diagnosis and monitoring of primary brain tumours, brain metastasis and acute ischaemic stroke all require invasive, burdensome and costly diagnostics, frequently lacking adequate sensitivity, particularly during disease monitoring. Monocytes are known to migrate to damaged tissues, where they act as tissue macrophages, continuously scavenging, phagocytizing and digesting apoptotic cells and other tissue debris. We hypothesize that upon completion of their tissue-cleaning task, these tissue macrophages might migrate via the lymph system to the bloodstream, where they can be detected and evaluated for their phagolysosomal contents. We discovered a blood monocyte subpopulation carrying the brain-specific glial fibrillary acidic protein in glioma patients and in patients with brain metastasis and evaluated the diagnostic potential of this finding. Blood samples were collected in a cross-sectional study before or during surgery from adult patients with brain lesions suspected of glioma. Together with blood samples from healthy controls, these samples were flowing cytometrically evaluated for intracellular glial fibrillary acidic protein in monocyte subsets. Acute ischaemic stroke patients were tested at multiple time points after onset to evaluate the presence of glial fibrillary acidic protein-carrying monocytes in other forms of brain tissue damage. Clinical data were collected retrospectively. High-grade gliomas (*N* = 145), brain metastasis (*N* = 21) and large stroke patients (>100 cm^3^) (*N* = 3 versus 6; multiple time points) had significantly increased frequencies of glial fibrillary acidic protein+CD16+ monocytes compared to healthy controls. Based on both a training and validation set, a cut-off value of 0.6% glial fibrillary acidic protein+CD16+ monocytes was established, with 81% sensitivity (95% CI 75–87%) and 85% specificity (95% CI 80–90%) for brain lesion detection. Acute ischaemic strokes of >100 cm^3^ reached >0.6% of glial fibrillary acidic protein+CD16+ monocytes within the first 2–8 h after hospitalization and subsided within 48 h. Glioblastoma patients with >20% glial fibrillary acidic protein+CD16+ non-classical monocytes had a significantly shorter median overall survival (8.1 versus 12.1 months). Our results and the available literature, support the hypothesis of a tissue-origin of these glial fibrillary acidic protein-carrying monocytes. Blood monocytes carrying glial fibrillary acidic protein have a high sensitivity and specificity for the detection of brain lesions and for glioblastoma patients with a decreased overall survival. Furthermore, their very rapid response to acute tissue damage identifies large areas of ischaemic tissue damage within 8 h after an ischaemic event. These studies are the first to report the clinical applicability for brain tissue damage detection through a minimally invasive diagnostic method, based on blood monocytes and not serum markers, with direct consequences for disease monitoring in future (therapeutic) studies and clinical decision making in glioma and acute ischaemic stroke patients.

## Teaser

Van den Bossche *et al.* report a blood monocyte subset carrying brain-specific proteins and that relatively increases in case of brain tissue damage such as in glioma, brain metastases and stroke, which can be used to detect and monitor disease. Moreover, these monocytes predict survival in glioblastoma patients at presentation.

## Graphical Abstract

**Graphical Abstract:**
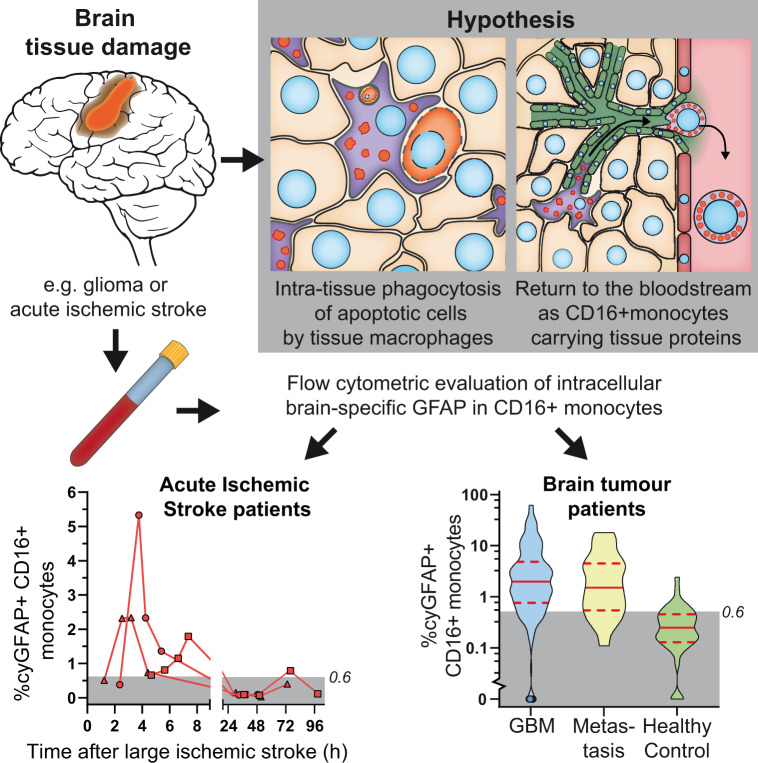

## Introduction

Improving diagnosis and monitoring of disease is an important goal in medicine. In brain tumour diagnostics, modern imaging techniques often lack sufficient sensitivity and specificity whereas tissue diagnosis requires invasive surgery with associated risks. Gliomas are aggressive brain tumours with a median survival of ∼12–15 months (Stupp *et al.*, 2009; Johnson and O’Neill, 2012). Diagnosis and staging depend on histopathological and molecular analysis of the tumour tissue (Louis *et al.*, 2016), which requires surgical resection or tumour biopsy. MRI imaging lacks grading capabilities and has limited use in disease monitoring (Jackson *et al.*, 2001; Boonzaier *et al.*, 2015; Thust *et al.*, 2018). Therefore, approaches using blood or cerebrospinal fluid, i.e., ‘liquid biopsies’ are considered as potentially more attractive methods because they are less invasive, less expensive, and can be applied more frequently. Promising techniques like spectrometry, circulating tumour cells, extracellular vesicles and serum markers have been demonstrated to be potentially useful tools in diagnostics, but they have not yet delivered broadly applicable methods to support clinical decision making in glioma and brain metastasis (Verheul *et al.*, 2017; Zachariah *et al.*, 2018; Butler *et al.*, 2019).

A similar need for blood-based biomarkers exists for Acute Ischemic Strokes (AIS), where diagnostic procedures are based on radiological imaging. Blood-based diagnostics for early size-estimation and treatment effect evaluation applicable in the acute phase (<24 h after onset) are lacking (Ahmad *et al.*, 2012; Hasan *et al.*, 2012). Various serum biomarkers have been explored, but clinicians and researchers remain dependent on the low sensitivity of CT or the time-consuming and rarely immediately available MRI imaging (Mullins *et al.*, 2002; Vilela and Rowley, 2017; Zhang and Liang, 2019).

We here report a novel blood monocyte-based diagnostic strategy, discovered while studying monocyte-macrophage trafficking in gliomas. Blood monocytes are patrolling myeloid cells that are assumed to migrate to sites of tissue damage such as in glioma and AIS in response to brain tissue damage; they act in addition to the brain-resident yolk-sac derived microglia (Yang *et al.*, 2011; Ritzel *et al.*, 2015). Several blood monocyte subpopulations can be distinguished, based on CD14 and CD16 expression, including classical (CD14+CD16−), intermediate (CD14+CD16+) and non-classical (CD14−CD16+) monocytes (Ziegler-Heitbrock *et al.*, 2010). CD16+ monocytes significantly increase in the bloodstream upon disrupted tissue homeostasis such as in inflammation, sepsis and cancer (Fingerle *et al.*, 1993; Wong *et al.*, 2012). Monocyte-derived macrophages (CD68+) (Micklem *et al.*, 1989; Uhlen *et al.*, 2015) can be detected in almost all tissues and are the assumed end-stage cells in the monocyte-macrophage lineage. However, they can also be detected in lymph vessels (Almeida *et al.*, 2001) as well as in the thoracic duct during inflammation, from where lymph drains into the bloodstream (Bell and Botham, 1982; Lemaire *et al.*, 1998). Furthermore, tissue macrophages (TiMas) have been described to contain tissue-specific proteins in tumour tissues and in draining lymph nodes in various diseases (Bell and Botham, 1982; van Zwam *et al.*, 2009; Faber *et al.*, 2012). These TiMas continuously phagocytize and digest apoptotic cells and tissue debris. We hypothesize that when these TiMas have fulfilled their local tissue-cleaning task, they migrate via lymph vessels to draining lymph nodes and, consequently, at least in part they recirculate to the bloodstream, where they can be detected by flow cytometry and evaluated for the contents of their phagolysosomes. If this recirculation concept is correct, a potential protein for detection in these recirculated TiMas is a glial fibrillary acidic protein (GFAP), which is primarily expressed in astrocytes and gliomas (Jacque *et al.*, 1978; Uhlen *et al.*, 2015). It can be found as a soluble protein in serum but with limited sensitivity and specificity for disease detection, and it has not been described to be present in blood leukocytes (Wunderlich *et al.*, 2006; Jung *et al.*, 2007).

The myelin-specific proteolipid protein (PLP1) and glioma-specific mutated proteins EGFRvIII and IDH1R132H, have not been found in serum but are other highly tissue- or tumour-specific proteins (Moscatello *et al.*, 1995; Klugmann *et al.*, 1997; Yan *et al.*, 2009; Uhlen *et al.*, 2015). Here, we describe for the first time a large-scale prospective study evaluating the applicability of a novel diagnostic strategy based on the detection of blood monocytes, carrying brain-specific proteins intracellularly in glioma, brain metastases and AIS.

## Materials and methods

### Study design

Blood (10 mL) was drawn from adult (>18 years) glioma-suspected patients at the outpatient clinic and/or during surgical resection of the tumour before surgery was finished and before the formal pathology diagnosis was made as well as from AIS patients at various time points in the emergency department and during hospitalization. Flow cytometric data analysis was performed blinded to clinical information. The clinical characteristics were retrospectively and independently assessed. As no comparable diagnostic reference standard exists, flow cytometric results were compared to pathological diagnosis, radiological characteristics and survival data. Tumour size was based on the average of the broadest diameter of the tumour in three directions on MRI or CT with contrast. IDH1 and EGFR status were determined by the Department of Pathology. Because of its novelty, this study was performed as an initial exploratory study. Our aim was to include 300 glioma patients, because of the incidence of lower-grade gliomas and brain metastasis among glioma-suspected patients in our hospital, aiming for a minimum of 25 patients per histopathological grade (grades II, III and IV gliomas and brain metastasis; with exception of the rare WHO grade I gliomas).

Blood was taken at multiple time points from adult AIS patients within 6 h after stroke onset, admitted to the Erasmus MC. We aimed to evaluate nine AIS patients, based on the established sensitivity and specificity in the first series of glioma-suspect patients.

### Participants

Patients with suspicion of primary glioma at the Erasmus MC Neurosurgery outpatient clinic between April 2011 and March 2018, were randomly asked written participation consent. The histopathological grade was defined according to the 2016 WHO classification of tumours of the Central Nervous System criteria (Louis *et al.*, 2016). Grading performed prior to 2016 was updated if genetic tumour data were available. Progression-free survival was calculated using the blood sampling date and first radiological confirmation of disease progression based on established criteria (Wen *et al.*, 2010).

For adult AIS patients admitted to the Erasmus MC with clinical and radiological diagnosis of AIS who were within 6 h from the onset, were retrospectively asked for written consent. Patients were excluded if there were contraindications for MRI, in case of cerebral bleeding during revascularization, or when they used prior immunosuppressive medication. Blood was drawn at several time points. The infarct size was assessed on Day 5 or at discharge if earlier, using a dedicated MRI protocol (DWI, T2w and 3 D FLAIR).

All participating patients provided written informed consent for study participation. Both studies were approved by the local Medical Ethics Committee of Erasmus MC Rotterdam in compliance with the Declaration of Helsinki (Glioma study MEC 221.520/2002/262 and Acute ischaemic stroke-study NL48968.078.14).

### Tumour dissociation and immunocytochemistry

Patient-derived tumour tissue was dissociated as described previously (Balvers *et al.*, 2013). After dissociation, tumour cells were treated with erythrocyte lysis buffer, cytospins were prepared using a Shandon Centrifuge and fixed in −20^**°**^C acetone. Samples were treated with Protein Block (Dako, Glostrup, Denmark) before staining with anti-CD68 (mouse clone KP-1, Dako) and anti-GFAP (rabbit polyclonal, Dako). ALEXA-488 rabbit-anti-mouse and ALEXA-546 goat-anti-rabbit IgG (H + L)(Life Technologies Europe, Bleiswijk, Netherlands) were added as secondary antibodies. Slides were then stained with DAPI (Vectashield, Vector Laboratories, Burlingame, CA). Imaging was performed at room temperature on a Leica DMRB Binocular microscope, with a Hamamatsu Orca-ER camera. ImageJ software (Rasband, W.S., ImageJ, U.S. National Institute of Health, Bethesda, MD) was used for image processing.

### Flow cytometry studies

For immunophenotypic studies, our TiMaScan flow cytometry antibody panel (for details see: Supplementary Table 1) was used. Blood was collected in EDTA tubes and was processed within 8 h after drawing. The material was first processed with bulk lysis (NH_4_CL lysis buffer) to reduce the amount of erythrocytes and then directly stained for membrane markers. Intracellular staining was performed using Fix&Perm (Nordic Mubio, Susteren, NL), sample acquisition was performed on FACSCanto II instruments (BD Biosciences, San José, CA) and Infinicyt software v1.8 (Cytognos, Salamanca, Spain) was used for data analysis. Samples were processed and analysed freshly within 8 h; they were not frozen/thawed or stored in between the various steps of the flow cytometry protocols. Debris and doublets were identified based on FSC-Area plus FSC-Height and FSC-Area plus SSC-Area. Monocytes were identified by consecutive gating steps using SSC plus FSC-Area, SSC-Area plus CD45, HLA-DR plus CD300e, CD300e plus CD14 and CD14 plus CD16; subsequent monocyte subsetting was performed based on CD14 plusCD16 and HLA-DR plus CD14 (for gating strategy, see: Supplementary Fig. 1). GFAP- and PLP1-positivity in monocyte subsets was evaluated, based on the fluorescent background in lymphocytes. All the above protocols and related quality control were performed according to EuroFlow protocols (www.EuroFlow.org) (Kalina *et al.*, 2012; van Dongen *et al.*, 2012).

### Serum GFAP

Fresh blood was centrifuged (10 000 rpm, 5 min, room temperature) and serum was then stored at −20^**°**^C. Measurements were performed after the collection of all samples was completed. GFAP serum levels were evaluated twice in two separately stored samples per patient, according to the manufacturer’s instructions with the GFAP Human Elisa-kit (Biovendor, Brno, CZ).

### Statistical analysis

All statistical analyses were performed using SPSS Statistics v24 (IBM, Armonk, NY). Patients with missing data were excluded from analyses. Data with normal distribution were analysed using one-way ANOVA followed by Tukey’s multiple comparison test. The Mann–Whitney *U* test was used for non-Gaussian distributed data. Differences were considered statistically significant when *P* < 0.05. The Bonferroni correction was applied to repeated analyses.

Receiver operating characteristic curves were built based on a training dataset, consisting of randomly selected patients (33%). Cut-off (CO) values were determined with the training set using a method by Unal (2017) identifying the CO-point at which sensitivity and specificity are closest. These CO-values were then applied to the validation dataset (remaining 66% of patients) and all healthy controls. The *χ*^2^ test for independence was then used to assess test performance.

Cox-regression was used to evaluate continuous values and overall survival (OS) and progression-free survival. If significant (*P* < 0.05), the median survival at each CO-point for both the test-positive and test-negative group was calculated and compared. The CO-points were set when the maximum difference between median survival in each patient group was reached and were then evaluated by log-rank test.

### Data availability

The data that support the findings of this manuscript are available from the corresponding author, upon reasonable request.

## Results

### Patient characteristics

Between June 2011 and March 2018, 260 blood samples from 228 glioma-suspect patients that met the inclusion criteria and test-quality, were evaluated for GFAP+ monocytes (Supplementary Fig. 2A, Patient flow chart). Most patients were men (62%) and histopathological diagnoses included glioma WHO grade I, oligodendroglioma, diffuse astrocytoma, glioblastoma (GBM) and brain metastasis (Table 1). Blinded clinical evaluation was performed at least 6 months after flow cytometric evaluation of GFAP+ monocytes.

**Table 1:** Summary of clinical characteristics of glioma study participants

	*N* per time point		% **male**	**Median** **age**^a^	**% of multiple samples** ^b^	**Median** **FU**	**Median** **OS**^c^	**Median** **PFS**^d^	**Mean** **tumour size**	**Median** **KPS**^e^	**% Prior dexamethasone use** ^f^	Mean dexa- methasone dose	Cerebral lesions
	Pre-operative	Per-operative	%	Year range	%	months	months	months	cm		%	mg/day median	Average nr
WHO I	2	0	50	57	0	55			36	70	50	4.0 *4*	1
				40.5–73.5									
Diffuse astrocytoma	19	9	68	51.2	7	45	22	16	28	90	27,3	2.0 *0*	1
				25.1–73.1									
Oligodendroglioma	14	18	66	55.1	3	56	39	29	35	90	56,3	6.5 4	1,2
				35.2–76.4									
GBM	104	41	66	62.8	17	11	9	6,6	35	90	65,5	5.5 *4*	1,1
				25.1–81.5									
Metastasis	21	0	40	61.1	0	14	14	9,1	27	90	76,2	6.1 *8*	1
				37.8–78.3									
Healthy control	38		40	51.5	0					100	0	6.1 *8*	
				29.8–86.9									
Total^h^	160	68	62.1	60.6	12	14	15	7,7	34	90	53,4	5.3 4	1,1
				25.1–81.5									

FU = follow-up; OS = overall survival; PFS = progression free survival; KPS: Karnofsky Performance Score; mo; months; NOS = not-otherwise specified; WHO = World Health Organization.

a At the time of first blood sampling for flow cytometric analyses.

b Percentage of patients that underwent a second blood sampling.

c In case patients deceased.

d In case patients proved to have radiologically confirmed tumour recurrence.

e Karnofsky Performance Score at the first blood sampling for flow cytometric analyses.

f Patients that had used dexamethasone in the 3 days before blood sampling.

h Total does not include healthy controls.

Blank fields had too limited numbers for accurate calculation of the corresponding value.

Additionally, nine AIS patients (Table 2) were evaluated between July 2014 and August 2015 with subsequent GFAP+CD16+ monocyte measurements from presentation until hospital discharge. No adverse events due to blood drawing were reported (Supplementary Fig. 2B, Patient flow chart).

**Table 2:** Summary of clinical characteristics of AIS study participants

	Gender	Age	Hemisphere of lesion	NIHSS	Infarct size (cm^3^)	Thrombolysis IV	Thrombectomy	Infarct type
AIS001	M	39	Left	1	16.7	Yes	No	PACI
AIS002	F	75	Left	22	25.2	Yes	No	TACI
AIS004	M	66	N.A.	0	0.1	Yes	No^a^	LACI
AIS005	F	79	Right	18	191.2	No	Yes	TACI
AIS007	M	75	N.A.	0	0	Yes	No	LACI
AIS008	M	63	N.A.	4	96.1	No	Yes^b^	PACI
AIS010	F	73	Left	7	33.2	No	Yes^b^	PACI
AIS013	M	68	N.A.	24	219.1	Yes	No	TACI
AIS014	M	69	N.A.	13	120	Yes	Yes^b^	TACI
Total	67% M	Median 69 years				67%	44%	

a Reperfusion not obtained.

b Reperfusion obtained.

LACI = lacunar infarction; NIHSS = NIH Stroke Scale; PACI = partial anterior circulation infarct; TACI = total anterior circulation infarct.

### Monocytes and macrophages carrying brain-specific proteins

**Figure 1:**
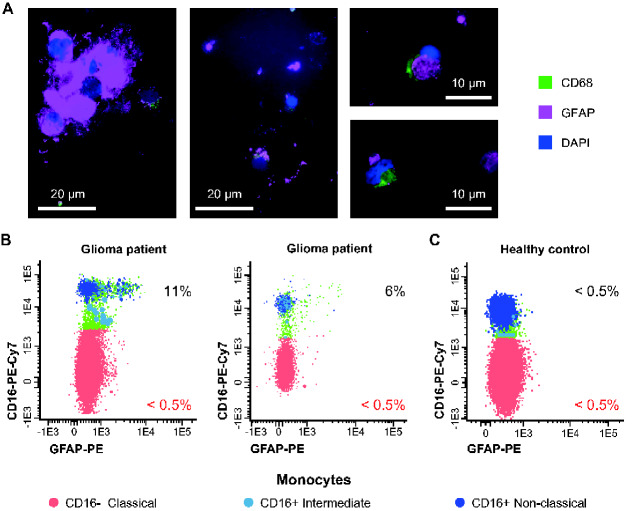
**Detection of GFAP+ macrophages in brain tissue and in monocytes from patients suspected of glioma.** (**A**) Dissociated glioma tissue contains CD68+ (green) and GFAP+ (magenta) cells. (**B**) Flow cytometric dot plots of blood monocytes from two patients suspected of glioma lesions and their GFAP expression levels. (**C**) Flow cytometric dot plot of GFAP expression in monocyte subsets in a representative example of a healthy control. White bar represents scale of the microscopy images. Percentage of GFAP+ cells (**B, C**). Dot plots show classical (red), intermediate (light blue) and non-classical (dark blue) monocytes.

Immunocytochemical staining of dissociated gliomas revealed GFAP-carrying (GFAP+) CD68+ cells (Fig. 1A). Also, flow cytometric analysis of glioma patients’ blood revealed a small population of GFAP+ intermediate monocytes and GFAP+ non-classical monocytes (Fig. 1B), while relatively very limited GFAP+ classical monocytes were found in the blood of healthy controls (Fig. 1C). The intermediate and non-classical monocyte populations, both expressing CD16 (CD16+ monocytes), have a significantly larger fraction of GFAP+ cells in 228 brain tumour patients than in healthy controls (*P* < 0.001) (Supplementary Fig. 3A). Absolute numbers of GFAP+CD16+ monocytes per millilitre were also significantly increased but to a lesser extent than the relative numbers (Supplementary Fig. 3B). Many glioma patients receive dexamethasone to decrease peri-tumoral oedema and tumour mass effect. In patients using dexamethasone absolute leukocyte numbers were increased (*P* < 0.001) (Supplementary Fig. 4A), while absolute numbers of non-classical (*P* < 0.005) and CD16+ (*P* < 0.01) monocytes, lymphocytes (*P* < 0.01) and dendritic cells (*P* < 0.01) were significantly decreased compared to patients without dexamethasone (Supplementary Fig. 4B); no significant differences were found in relative numbers of GFAP+CD16+ monocytes (*P* = 0.070) (Supplementary Fig. 4C).

Soluble GFAP has been previously described to be elevated in the plasma of GBM patients (Jung *et al.*, 2007; Kiviniemi *et al.*, 2015) and possibly applicable in a clinical setting with a sensitivity of 86% and 85% for these high-grade tumours. As soluble GFAP might be taken up by monocytes from plasma through pinocytosis, plasma GFAP might be the origin of the GFAP present in CD16+ monocytes. Therefore, we evaluated whether a relationship existed between plasma GFAP levels and GFAP+CD16+ monocytes. No correlation was found between these parameters (astrocytoma *P* = 0.892, oligodendroglioma *P* = 0.636, GBM *P* = 0.065) (Supplementary Fig. 5). In our series, we found that plasma GFAP had a sensitivity of 18.2%, 35.3% and 57.5% for astrocytoma (*N* = 11), oligodendroglioma (*N* = 17) and GBM (*N* = 40), respectively. Additionally, we compared the mean fluorescence intensity of GFAP-PE in the classical monocytes of patients with a low (<0.001 µg/l) and high (>0.22 µg/l) plasma GFAP concentration. There was no difference in GFAP mean fluorescence intensity between high and low plasma GFAP patients (*P*** **=** **0.143).

The GFAP-positivity of the tumour and the timing of the blood sampling, before or during surgery (Supplementary Fig. 6A and B) did not affect GFAP+CD16+ monocytes. However, tumour volume correlated to the percentage of GFAP+CD16+ monocytes for both GBM (*r* = 0.215; *P* = 0.010) and brain metastasis (*r* = 0.504; *P* = 0.010) (Supplementary Fig. 6C).

The myelin-specific protein PLP1 was also detected in CD16+ monocytes and was significantly increased in GBM patients (*P* = 0.003) (Supplementary Fig. 7A and B). We also found CD16+ monocytes carrying both GFAP and PLP1 (0.0–3.4%; *N* = 65) (Supplementary Fig. 7C). Moreover, a significant correlation between the relative presence of GFAP+ and PLP1+CD16+ monocytes in GBM patients was found (*r* = 0.516, *P* < 0.001) (Supplementary Fig. 7D). However, both the degree and frequency of PLP1 expression were less prominent compared to GFAP and were only significantly increased in GBM (Supplementary Fig. 7B). Consequently, we focused on using GFAP as a diagnostic marker in our TiMaScan assay (Supplementary Table 1).

In contrast to GFAP and PLP1, the mutated proteins IDH1R132H and EGFRvIII were not reliably detected with the applied antibodies in blood monocytes, as we found no relationship with the absence or presence of these mutations in the evaluated tumours (Supplementary Fig. 7E and F).

**Figure 2:**
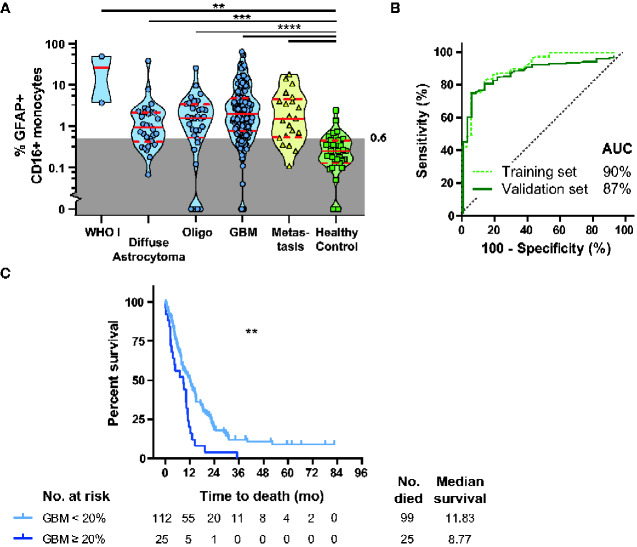
**Distribution of GFAP+CD16+ monocytes in blood in brain lesions versus healthy controls and relation to OS in GBM patients.** (**A**) Violin plot with median, quartiles and individual percentages of GFAP+CD16+ monocytes in blood of patients with different types of glioma, brain metastasis, other brain tumours and healthy controls (Kruskal–Wallis). (**B**) ROC curve with area under the curve for presence of a brain lesion, with GFAP+CD16+ monocytes as predictor in the training set (green dotted line (*N* = 82) and validation set (dark green line, *N* = 157). (**C**) Survival plot of GBM patients with GFAP+CD14−CD16+ monocytes below (light blue) or over 20% (dark blue) of GFAP-positivity. Ticks mark censored cases, Log-rank test. AUC = area under the curve; Oligo = oligodendroglioma; ROC = receiver operating characteristic; ** *P* < 0.01, *** *P* < 0.005, **** *P* < 0.001.

Per histopathological grade a significantly increased relative number of GFAP+CD16+ monocytes was found compared to healthy controls in diffuse astrocytoma (*P* < 0.005), oligodendroglioma (*P* < 0.001), GBM (*P* < 0.001) and brain metastasis (*P* < 0.001) (Fig. 2A). receiver operating characteristic-curve analyses of randomly selected patients (33%; *N* = 82) of various glioma grades and brain metastasis, showed a high accuracy area under the curve of 90% for the percentage CD16+GFAP+ monocytes predicting for a brain lesion. A validation set (*N* = 157) showed a very similar accuracy (area under the curve of 87%) (Fig. 2B). Based on the training dataset an optimal CO-value of 0.6% GFAP+CD16+ monocytes were established to predict for the presence of a brain lesion. This was then applied to the validation set and showed a sensitivity of 81% (95% CI 75–87%) and specificity of 85% (95% CI 80–90%) (Pearson’s chi-square(1) = 42.83, *P* < 0.001) (Supplementary Fig. 9A for cross-tabulation of the test results).

### Impact of GFAP+ monocyte frequencies on patient overall survival

GFAP+CD16+ monocyte relative numbers were evaluated for their relationship with OS and progression-free survival in glioma patients. Survival of the glioma patients in our cohort matched with those reported in the literature (Supplementary Fig. 8) (Johnson and O’Neill, 2012). Overall, no direct association was found between progression-free survival and GFAP+ monocyte subset counts. However, in patients with GBM, there was a significant inverse association with OS. Patients with >20% GFAP+CD16+CD14− monocytes had significantly decreased median OS, 8.8 months (*N* = 25) versus 11.8 months (*N* = 112) (Mantel–Cox: Chi-square 10.62(1), *P* = 0.001) (Fig. 2C). After adjustment for possible confounding factors as gender, age and Karnofsky Performance Score at sampling and tumour size, the statistical difference between the two groups remained significant (Cox-regression: chi-square 29.24(5), hazard ratio = 1.967, *P* = 0.018), Moreover, also brain metastasis, albeit not significant, demonstrated a decreased OS in case of high GFAP+CD16+ monocytes frequencies (log-rank test chi-square = 3.218, *P* = 0.73) (Supplementary Fig. 9B).

### Acute brain damage and GFAP+CD16+ monocytes

**Figure 3:**
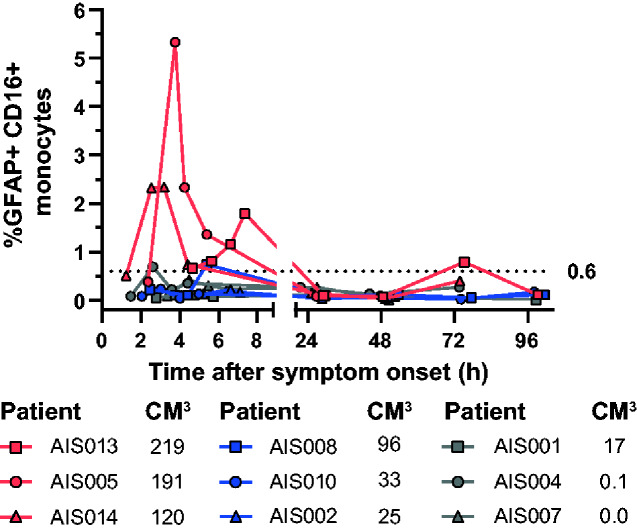
**Monitoring of percentage of GFAP+CD16+ monocytes in blood during the first 96 h following onset of stroke symptoms.** Relative numbers of GFAP+CD16+ monocytes in blood are shown for 9 AIS patients after stroke onset, coloured differently according to the size of their corresponding ischaemic lesion (in cm^3^): red ≥100 cm^3^; blue and grey: <100 cm^3^. The dotted line represents the 0.6% GFAP+CD16+ monocyte CO.

In AIS patients, we also found GFAP+CD16+ monocytes increased, while PLP1+CD16+ monocytes remained below 0.3% within the first 48 h. Patients with lesions over 100 cm^3^ (AIS005, AIS013 and AIS014) demonstrated at multiple time points above 0.6% GFAP+CD16+ monocytes, all within 8 h after onset. In patient AIS008 (96.1 cm^3^) and AIS001 (0.1 cm^3^), only one-time point reached above 0.6% GFAP+CD16+ monocytes, but remained below 1%. All other patients with lesions ranging from 0 cm^3^ to 33.2 cm^3^ did not reach 0.6%. After 8 h, only one sample reached over 0.6% (Fig. 3).

## Discussion

Our results demonstrate brain tissue-specific proteins such as GFAP and PLP1 to be present in both TiMas and subsets of blood monocytes, especially in diseases with brain tissue-damage such as glioma, brain metastases and AIS. The here used, 8-colour flow cytometry assay to detect these tissue-specific protein-carrying monocytes, is relatively cheap, fast (processing and analysis within 3 h) and can thereby be easily applied in most hospitals.

In our large series of glioma suspect patients, we found a significant increase in the percentage of GFAP-carrying CD16+ monocytes in all glioma grades and brain metastases. Since no significant differences between pathological grades were observed, the measurement of GFAP-carrying CD16+ monocytes cannot be used to stratify for glioma grades. However, it demonstrates its sensitivity for detection and quantitation of brain tissue damage in general. The absolute counts of GFAP+CD16+ monocytes in the blood, also showed an increase in glioma and brain metastasis patients, but these absolute counts are relatively low and quit variable (median 31 cells/µl, range 0.6–1439 cells/µl), most likely because these counts are affected by confounding factors such as, immune cell kinetics, sample processing and medication. For instance, dexamethasone, often prescribed to brain tumour patients, decreases monocyte numbers (Schuld *et al.*, 2001) but had no significant effect on the relative presence of GFAP+CD16+ monocytes, supporting the robustness and translatability of the here presented novel diagnostic strategy for the detection of brain tissue damage as caused by glioma, brain metastasis and AIS.

A large-scale evaluation of these cells in 228 glioma-suspect patients demonstrated that for the detection of a cerebral lesion sensitivity of 81% and specificity of 85% can be reached with a CO of 0.6% GFAP+CD16+ monocytes. Although there was no direct association with the tumours positivity for GFAP and there only was an association with size in GBM, these results suggest that the GFAP in these monocytes is derived from both the tumour and the surrounding damaged brain tissue. We also found an inverse correlation with GBM patients’ OS and possibly brain metastasis patients’ OS, although the numbers of metastasis patients were too low to reach a conclusion. This suggests that GFAP+ monocyte numbers might reflect various tumour factors, such as size and speed of growth. Moreover, we also demonstrated that multiple different tissue-specific proteins can be detected in a single analysis as we found both PLP1 and GFAP in single CD16+ monocytes in GBM patients. Although PLP1 did not improve sensitivity and specificity, GFAP and PLP1 levels were directly associated, as they probably originate from the same source. Consequently, our results combined with the available literature, make it hypothetically possible to evaluate the primary tumour of metastases with a primary-tumour-specific protein in GFAP+CD16+ monocytes.

Our study did not evaluate the precise origin of GFAP+CD16+ monocytes, but literature and the here presented results suggest that GFAP within these blood monocytes originates from brain tissue. While this is a concept, evidence supporting this concept can be found throughout the literature. For instance, other investigators have demonstrated that tissue-specific proteins can be found in macrophages in damaged tissue and draining lymph nodes (Mariani-Costantini *et al.*, 1991; van Zwam *et al.*, 2009; Faber *et al.*, 2012). Also, CD16+ monocytes are relatively increased in lymph vessels (Almeida *et al.*, 2001) and show delayed mobilization in tissue and blood upon tissue damage (Nahrendorf *et al.*, 2007; van den Bossche *et al.*, 2018). Tak *et al.* (2017) elegantly demonstrated this delay in mobilization between monocyte populations under homeostasis and suggest a gap in monocyte maturation.

One might speculate that GFAP in blood monocytes might have been taken up from the bloodstream, but no relation was observed between plasma GFAP levels and the frequency of GFAP+CD16+ monocytes and only a small percentage of CD16+ monocytes contains GFAP, instead of the entire monocyte population. Moreover, classical monocytes have previously been demonstrated to have an increased phagocytic/oxidative activity over CD16+ monocytes (Almeida *et al.*, 2001). Nevertheless, classical monocytes did not show an increased number of GFAP+ cells and no generally increased GFAP-expression (mean fluorescence intensity) in patients with increased plasma GFAP. These results strongly suggest limited or no uptake of plasma GFAP in any of the blood monocyte populations. Instead, these data support the hypothesis of TiMas carrying tissue-specific proteins and returning to the circulation.

Other tumour-specific proteins (e.g. EGFRvIII and IDH1R132H), which potentially could lead to pathological grading using CD16+ monocytes, were not detectable with the applied antibodies. Either because of low-level expression or over-digestion of the proteins after phagocytosis into such small protein fragments that they are not detectable by the applied single-epitope monoclonal antibodies (Delamarre *et al.*, 2005). In this context, it should be noted that the applied anti-GFAP antibody is a (multi-epitope) polyclonal antibody, which increases the chance of detection, even if part of the detected protein epitopes are damaged by digestion in the phagolysosomes.

The CO for GFAP+CD16+ monocytes as established in glioma patients was applied to AIS patients and could clearly discriminate between large (>100 cm^3^) and small infarct sizes within the first 8 h upon arrival at the ER, which is in contrast to other serum biomarkers, which take 48 h to become detectable (Wunderlich *et al.*, 2006). Infarct size evaluation within this early timeframe can only be performed using MRI DWI, which is rarely available on short notice and is difficult in non-cooperating patients (Fiebach *et al.*, 2002; Mullins *et al.*, 2002; Zhang and Liang, 2019). Therefore, GFAP+CD16+ monocyte evaluation potentially is a candidate for patient evaluation in future AIS studies. PLP1 was hardly detected in AIS patients, as expected, since damage to the myelin sheaths is not expected in the acute phase after AIS in contrast to neuronal and astrocyte damage. Unfortunately, due to the large number of time points per patient directly after AIS onset, we were unable to expand the numbers to a larger group and thereby establish more exact GFAP+CD16+monocyte-values and kinetic patterns fitting with clinical outcome parameters.

## Conclusion

Here, we report for the first time on a blood monocyte-based strategy to detect brain tissue damage in both tumour-related (slow/chronic damage) and ischaemic brain lesions (acute damage). This method detects brain tissue damage caused by primary brain tumours, brain metastasis and large AIS and also predicts GBM patients’ OS at an early stage. This novel, fast, relatively cheap and minimally invasive (blood-based) technique can detect brain tissue damage, where current technologies lack accuracy and efficiency (Boonzaier *et al.*, 2015; Thust *et al.*, 2018; Zhang and Liang, 2019). Measurement of GFAP-carrying monocytes could thus be applied in clinical trials and decision making in glioma, brain metastases and AIS, thereby significantly changing diagnostics in neurology and oncology.

## Supplementary material

Supplementary material is available at *Brain Communications* online.

## Supplementary Material

fcaa215_Supplementary_Data

## Abbreviations

AUC =: area under the curve
AIS =: acute ischaemic stroke
CO =: cut-off
GBM =: glioblastoma
GFAP =: glial fibrillary acidic protein
OS =: overall survival
PFS =: progression-free survival

## Notes

*Members of the TiMaScan research group, visit http://www.TimaScan.org

## References

[fcaa215-B1] Ahmad O, Wardlaw J, Fau-Whiteley WN, Whiteley WN. Correlation of levels of neuronal and glial markers with radiological measures of infarct volume in ischaemic stroke: a systematic review. Cerebrovasc Dis 2012; 33: 47–54. 22133844 10.1159/000332810

[fcaa215-B2] Almeida J, Bueno C, Alguero MC, Sanchez ML, de Santiago M, Escribano L, et al Comparative analysis of the morphological, cytochemical, immunophenotypical, and functional characteristics of normal human peripheral blood lineage(-)/CD16(+)/HLA-DR(+)/CD14(-/lo) cells, CD14(+) monocytes, and CD16(-) dendritic cells. Clinical Immunol 2001; 100: 325–38. 11513546 10.1006/clim.2001.5072

[fcaa215-B3] Balvers RK, Kleijn A, Kloezeman JJ, French PJ, Kremer A, van den Bent MJ, et al Serum-free culture success of glial tumors is related to specific molecular profiles and expression of extracellular matrix-associated gene modules. Neuro Oncol 2013; 15: 1684–95. 24046260 10.1093/neuonc/not116 PMC3829587

[fcaa215-B4] Bell EB, Botham J. Antigen transport. I. Demonstration and characterization of cells laden with antigen in thoracic duct lymph and blood. Immunology 1982; 47: 477–87. 7129528 PMC1555548

[fcaa215-B5] Boonzaier NR, Piccirillo SG, Watts C, Price SJ. Assessing and monitoring intratumor heterogeneity in glioblastoma: how far has multimodal imaging come? CNS Oncology 2015; 4: 399–410. 26497327 10.2217/cns.15.20 PMC6083939

[fcaa215-B6] Butler HJ, Brennan PM, Cameron JM, Finlayson D, Hegarty MG, Jenkinson MD, et al Development of high-throughput ATR-FTIR technology for rapid triage of brain cancer. Nat Commun 2019; 10: 4501. 31594931 10.1038/s41467-019-12527-5 PMC6783469

[fcaa215-B7] Delamarre L, Pack M, Chang H, Mellman I, Trombetta ES. Differential lysosomal proteolysis in antigen-presenting cells determines antigen fate. Science 2005; 307: 1630–4. 15761154 10.1126/science.1108003

[fcaa215-B8] Faber TJ, Japink D, Leers MP, Sosef MN, von Meyenfeldt MF, Nap M. Activated macrophages containing tumor marker in colon carcinoma: immunohistochemical proof of a concept. Tumor Biol 2012; 33: 435–41. 10.1007/s13277-011-0269-z 22134871

[fcaa215-B9] Fiebach JB, Schellinger PD, Jansen O, Meyer M, Wilde P, Bender J, et al CT and diffusion-weighted MR imaging in randomized order: diffusion-weighted imaging results in higher accuracy and lower interrater variability in the diagnosis of hyperacute ischemic stroke. Stroke 2002; 33: 2206–10. 12215588 10.1161/01.str.0000026864.20339.cb

[fcaa215-B10] Fingerle G, Pforte A, Passlick B, Blumenstein M, Strobel M, Ziegler-Heitbrock HW. The novel subset of CD14+/CD16+ blood monocytes is expanded in sepsis patients. Blood 1993; 82: 3170–6. 7693040

[fcaa215-B11] Hasan N, McColgan P, Fau-Bentley P, Bentley P, Fau-Edwards RJ, Edwards R, Fau-Sharma P, Sharma P. Towards the identification of blood biomarkers for acute stroke in humans: a comprehensive systematic review. Br J Clin Pharmacol 2012; 74: 230–40. 22320313 10.1111/j.1365-2125.2012.04212.x PMC3630743

[fcaa215-B12] Jackson RJ, Fuller GN, Abi-Said D, Lang FF, Gokaslan ZL, Shi WM, et al Limitations of stereotactic biopsy in the initial management of gliomas. Neuro Oncol 2001; 3: 193–200. 11465400 10.1093/neuonc/3.3.193 PMC1920616

[fcaa215-B13] Jacque CM, Vinner C, Kujas M, Raoul M, Racadot J, Baumann NA. Determination of glial fibrillary acidic protein (GFAP) in human brain tumors. J Neurol Sci 1978; 35: 147–55. 624958 10.1016/0022-510x(78)90107-7

[fcaa215-B14] Johnson DR, O’Neill BP. Glioblastoma survival in the United States before and during the temozolomide era. J Neurooncol 2012; 107: 359–64. 22045118 10.1007/s11060-011-0749-4

[fcaa215-B15] Jung CS, Foerch C, Schanzer A, Heck A, Plate KH, Seifert V, et al Serum GFAP is a diagnostic marker for glioblastoma multiforme. Brain 2007; 130: 3336–41. 17998256 10.1093/brain/awm263

[fcaa215-B16] Kalina T, Flores-Montero J, van der Velden VH, Martin-Ayuso M, Bottcher S, Ritgen M; on behalf of the EuroFlow Consortium (EU-FP6, LSHB-CT-2006-018708), et al EuroFlow standardization of flow cytometer instrument settings and immunophenotyping protocols. Leukemia 2012; 26: 1986–2010. 22948490 10.1038/leu.2012.122 PMC3437409

[fcaa215-B17] Kiviniemi A, Gardberg M, Frantzen J, Parkkola R, Vuorinen V, Pesola M, et al Serum levels of GFAP and EGFR in primary and recurrent high-grade gliomas: correlation to tumor volume, molecular markers, and progression-free survival. J Neurooncol 2015; 124: 237–45. 26033547 10.1007/s11060-015-1829-7

[fcaa215-B18] Klugmann M, Schwab M, Fau-Puhlhofer A, Puhlhofer A, Fau-Schneider A, Schneider A, et al Assembly of CNS myelin in the absence of proteolipid protein. Neuron 1997; 18: 59–70. 9010205 10.1016/s0896-6273(01)80046-5

[fcaa215-B19] Lemaire LC, van Deventer SJ, van Lanschot JJ, Meenan J, Gouma DJ. Phenotypical characterization of cells in the thoracic duct of patients with and without systemic inflammatory response syndrome and multiple organ failure. Scand J Immunol 1998; 47: 69–75. 9467661 10.1046/j.1365-3083.1998.00265.x

[fcaa215-B20] Louis DN, Perry A, Reifenberger G, von Deimling A, Figarella-Branger D, Cavenee WK, et al The 2016 World Health Organization classification of tumors of the central nervous system: a summary. Acta Neuropathol 2016; 131: 803–20. 27157931 10.1007/s00401-016-1545-1

[fcaa215-B21] Mariani-Costantini R, Muraro R, Ficari F, Valli C, Bei R, Tonelli F, et al Immunohistochemical evidence of immune responses to tumor-associated antigens in lymph nodes of colon carcinoma patients. Cancer 1991; 67: 2880–6. 1709062 10.1002/1097-0142(19910601)67:11<2880::aid-cncr2820671129>3.0.co;2-a

[fcaa215-B22] Micklem K, Rigney E, Fau-Cordell J, Cordell J, Fau-Simmons D, Simmons D, et al A human macrophage-associated antigen (CD68) detected by six different monoclonal antibodies. Br J Haematol 1989; 73: 6–11. 2803980 10.1111/j.1365-2141.1989.tb00210.x

[fcaa215-B23] Moscatello DK, Holgado-Madruga M, Godwin AK, Ramirez G, Gunn G, Zoltick PW, et al Frequent expression of a mutant epidermal growth factor receptor in multiple human tumors. Cancer Res 1995; 55: 5536–9. 7585629

[fcaa215-B24] Mullins ME, Schaefer P, Fau-Sorensen AG, Sorensen A, Fau-Halpern EF, Halpern E, et al CT and conventional and diffusion-weighted MR imaging in acute stroke: study in 691 patients at presentation to the emergency department. Radiology 2002; 224: 353–60. 12147827 10.1148/radiol.2242010873

[fcaa215-B25] Nahrendorf M, Swirski FK, Aikawa E, Stangenberg L, Wurdinger T, Figueiredo JL, et al The healing myocardium sequentially mobilizes two monocyte subsets with divergent and complementary functions. J Exp Med 2007; 204: 3037–47. 18025128 10.1084/jem.20070885 PMC2118517

[fcaa215-B26] Ritzel RM, Patel AR, Grenier JM, Crapser J, Verma R, Jellison ER, et al Functional differences between microglia and monocytes after ischemic stroke. J Neuroinflammation 2015; 12:10.1186/s12974-015-0329-1 PMC4465481 26022493

[fcaa215-B27] Schuld A, Kraus T, Haack M, Hinze-Selch D, Zobel AW, Holsboer F, et al Effects of dexamethasone on cytokine plasma levels and white blood cell counts in depressed patients. Psychoneuroendocrinology 2001; 26: 65–76. 11070335 10.1016/s0306-4530(00)00039-1

[fcaa215-B28] Stupp R, Hegi ME, Mason WP, van den Bent MJ, Taphoorn MJ, Janzer RC, et al Effects of radiotherapy with concomitant and adjuvant temozolomide versus radiotherapy alone on survival in glioblastoma in a randomised phase III study: 5-year analysis of the EORTC-NCIC trial. Lancet Oncol 2009; 10: 459–66. 19269895 10.1016/S1470-2045(09)70025-7

[fcaa215-B29] Tak T, Drylewicz J, Conemans L, de Boer RJ, Koenderman L, Borghans JAM, et al Circulatory and maturation kinetics of human monocyte subsets in vivo. Blood 2017; 130: 1474–7. 28743715 10.1182/blood-2017-03-771261

[fcaa215-B30] Thust SC, van den Bent MJ, Smits M. Pseudoprogression of brain tumors. J Magn Reson Imaging 2018; 48: 571–89. 10.1002/jmri.26171 PMC6175399 29734497

[fcaa215-B31] Uhlen M, Fagerberg L, Hallstrom BM, Lindskog C, Oksvold P, Mardinoglu A, et al Proteomics. Tissue-based map of the human proteome. Science 2015; 347: 1260419. 25613900 10.1126/science.1260419

[fcaa215-B32] Unal I. Defining an optimal cut-point value in roc analysis: an alternative approach. Comput Math Methods Med 2017; 2017: 1–14. 10.1155/2017/3762651 PMC5470053 28642804

[fcaa215-B33] van den Bossche WBL, Rykov K, Teodosio C, Ten Have B, Knobben BAS, Sietsma MS, et al Flow cytometric assessment of leukocyte kinetics for the monitoring of tissue damage. Clin Immunol (Orlando, FL) 2018; 197: 224–30. 10.1016/j.clim.2018.09.014 30290225

[fcaa215-B34] van Dongen JJM, Lhermitte L, Bottcher S, Almeida J, van der Velden VHJ, Flores-Montero J; on behalf of the EuroFlow Consortium (EU-FP6, LSHB-CT-2006-018708), et al EuroFlow antibody panels for standardized n-dimensional flow cytometric immunophenotyping of normal, reactive and malignant leukocytes. Leukemia 2012; 26: 1908–75. 22552007 10.1038/leu.2012.120 PMC3437410

[fcaa215-B35] van Zwam M, Huizinga R, Melief MJ, Wierenga-Wolf AF, van Meurs M, Voerman JS, et al Brain antigens in functionally distinct antigen-presenting cell populations in cervical lymph nodes in MS and EAE. J Mol Med 2009; 87: 273–86. 19050840 10.1007/s00109-008-0421-4

[fcaa215-B36] Verheul C, Kleijn A, Lamfers MLM. Cerebrospinal fluid biomarkers of malignancies located in the central nervous system. Handb Clin Neurol 2017; 146: 139–69. 29110768 10.1016/B978-0-12-804279-3.00010-1

[fcaa215-B37] Vilela P, Rowley HA. Brain ischemia: CT and MRI techniques in acute ischemic stroke. Eur J Radiol 2017; 96: 162–72. 29054448 10.1016/j.ejrad.2017.08.014

[fcaa215-B38] Wen PY, Macdonald DR, Reardon DA, Cloughesy TF, Sorensen AG, Galanis E, et al Updated response assessment criteria for high-grade gliomas: response assessment in neuro-oncology working group. J Clin Oncol 2010; 28: 1963–72. 20231676 10.1200/JCO.2009.26.3541

[fcaa215-B39] Wong KL, Yeap WH, Tai JJ, Ong SM, Dang TM, Wong SC. The three human monocyte subsets: implications for health and disease. Immunol Res 2012; 53: 41–57. 22430559 10.1007/s12026-012-8297-3

[fcaa215-B40] Wunderlich MT, Wallesch C, Fau-Goertler M, Goertler M. Release of glial fibrillary acidic protein is related to the neurovascular status in acute ischemic stroke. Eur J Neurol 2006; 13: 1118–23. 16987165 10.1111/j.1468-1331.2006.01435.x

[fcaa215-B41] Yan H, Parsons DW, Jin G, McLendon R, Rasheed BA, Yuan W, et al IDH1 and IDH2 mutations in gliomas. N Engl J Med 2009; 360: 765–73. 19228619 10.1056/NEJMoa0808710 PMC2820383

[fcaa215-B42] Yang I, Han SJ, Sughrue ME, Tihan T, Parsa AT. Immune cell infiltrate differences in pilocytic astrocytoma and glioblastoma: evidence of distinct immunological microenvironments that reflect tumor biology. J Neurosurg 2011; 115: 505–11. 21663411 10.3171/2011.4.JNS101172

[fcaa215-B43] Zachariah MA, Oliveira-Costa JP, Carter BS, Stott SL, Nahed BV. Blood-based biomarkers for the diagnosis and monitoring of gliomas. Neuro Oncol 2018; 20: 1155–61. 29746665 10.1093/neuonc/noy074 PMC6071656

[fcaa215-B44] Zhang XH, Liang HM. Systematic review with network meta-analysis: Diagnostic values of ultrasonography, computed tomography, and magnetic resonance imaging in patients with ischemic stroke. Medicine 2019; 98: e16360. 31348236 10.1097/MD.0000000000016360 PMC6709059

[fcaa215-B45] Ziegler-Heitbrock L, Ancuta P, Crowe S, Dalod M, Grau V, Hart DN, et al Nomenclature of monocytes and dendritic cells in blood. Blood 2010; 116: e74-80. 20628149 10.1182/blood-2010-02-258558

