# Supplementary material for: Monocytes carrying GFAP detect glioma, brain metastasis and ischaemic stroke, and predict glioblastoma survival
Source: Brain Commun. 2020 Dec 26;3(1):fcaa215. doi: 10.1093/braincomms/fcaa215 (PMC7811761; doi:10.1093/braincomms/fcaa215)
Supplement: fcaa215_Supplementary_Data [file fcaa215_supplementary_data.pdf]

Supplementary Figure 1

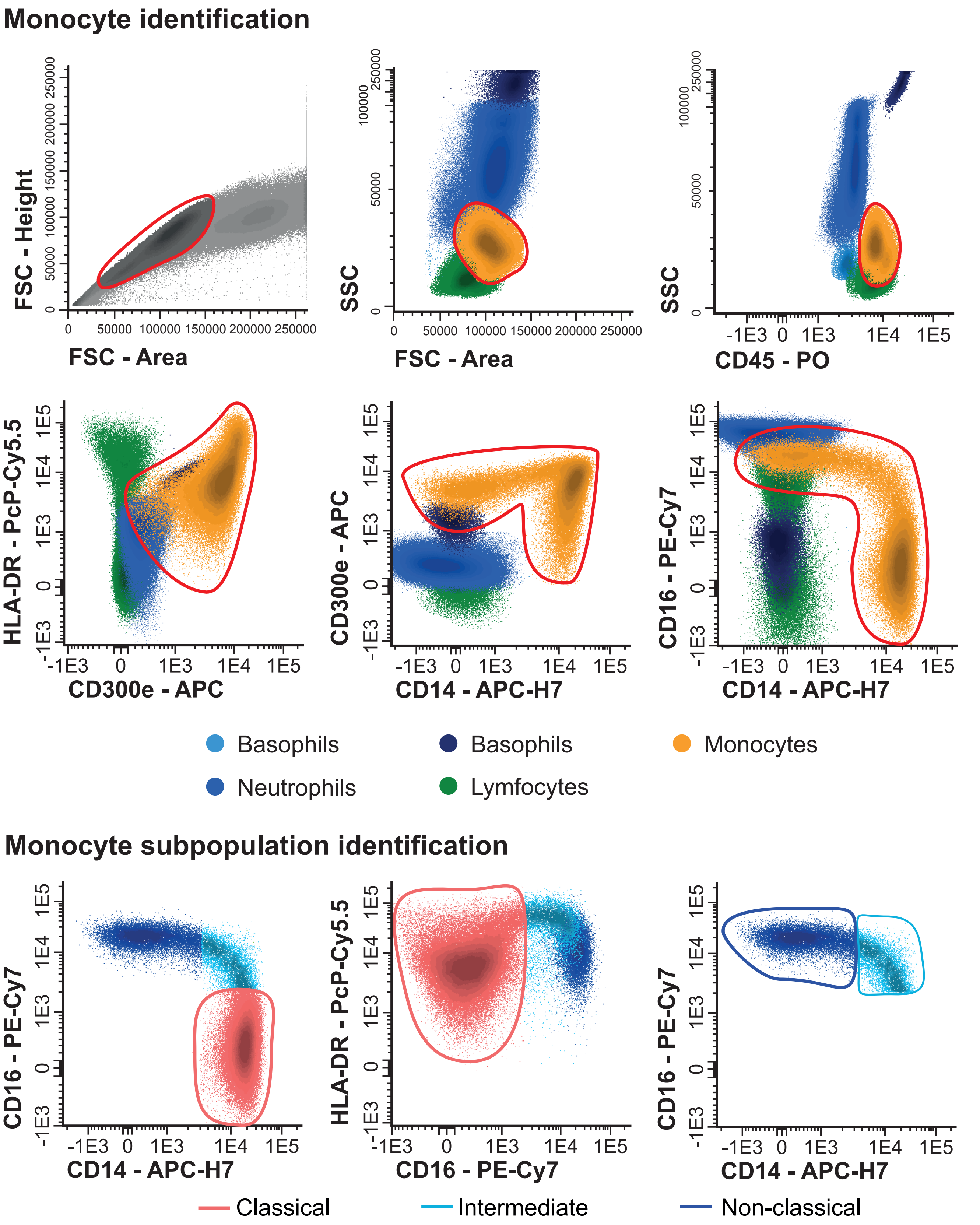

Supplementary Figure 1 Flow cytometric gating strategy used for the identification of monocyte subsets. The initial step of the flow cytometric identification is the removal of debris and doublets based on FSC-Area and FSC-Height. Followed by monocyte identification using consecutive gating steps; SSC plus FSC-Area, SSC-Area plus CD45, HLA-DR plus CD300e, CD300e plus CD14, and CD14 plus CD16; subsequent monocyte subsetting was performed based on CD14 plusCD16 and CD14 plus HLA-DR. After monocyte identification, subsets were identified using CD14 plus CD16 and HLA-DR plus CD14.

Supplementary Figure 2

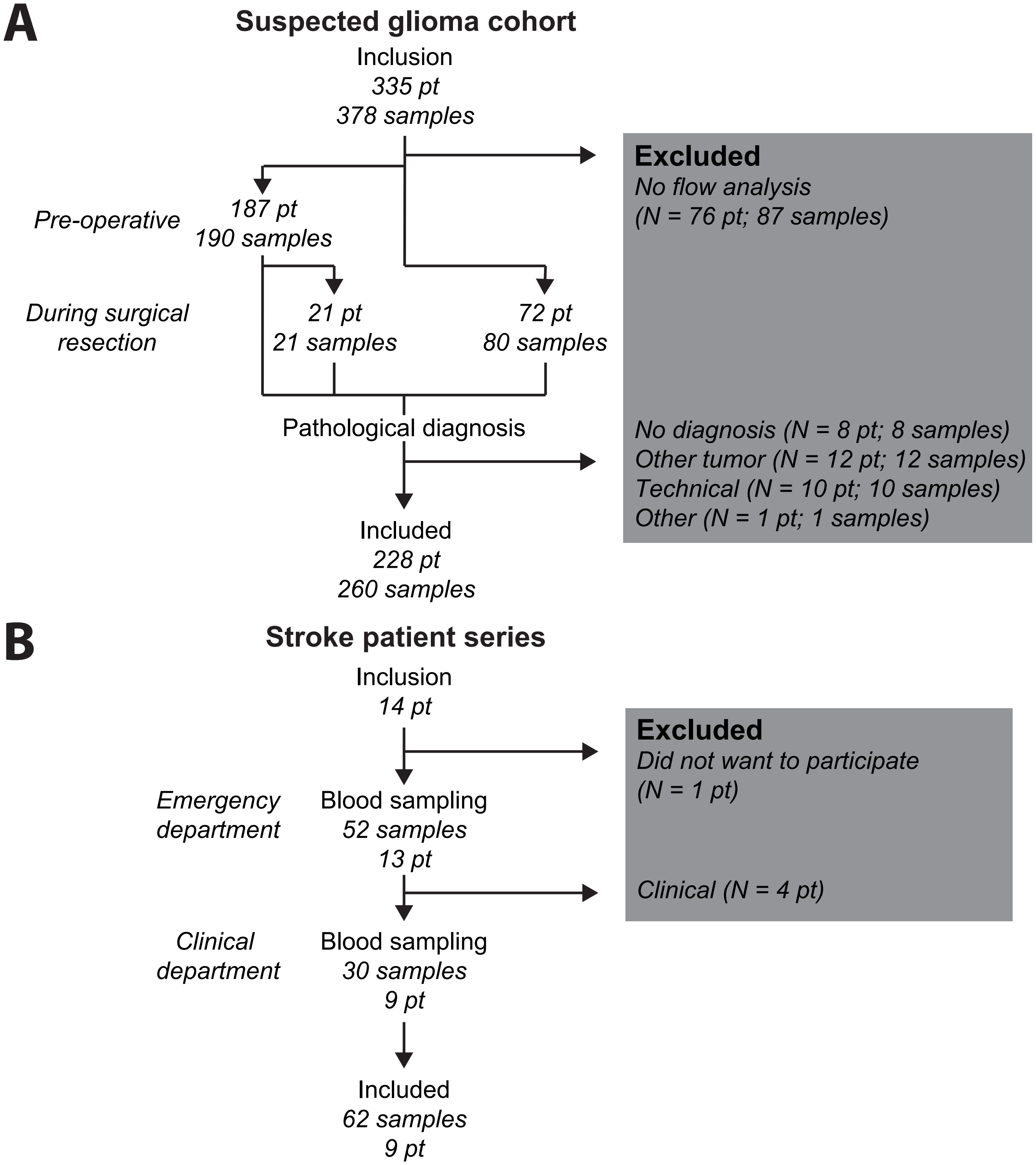

Supplementary Figure 2 Flow chart summarizing patients and samples included in both the Glioma and Stroke study. Glioma study patients provided either their first blood sample at the outpatient clinic (N=187) or during the surgical resection of their tumour (N=72). 21 patients provided a primary sample at the outpatient clinic and during surgery. Reasons for exclusion consisted of lack of diagnosis (as no tissue was obtained or no diagnosis could be made on the obtained tumour material). Other tumour consisted of one lymphoma and one meningioma. Technical reasons for exclusion were improper acquisition of data, technical failure of the flow cytometer during sample acquisition or data file error caused by the transfer of files to the central servers. All Stroke study patients provided their first sample at their admittance to the Emergency Room. Reasons for exclusion were not willing to participate (N=1) and not meeting the clinical inclusion criteria (N=4).

Supplementary Figure 3

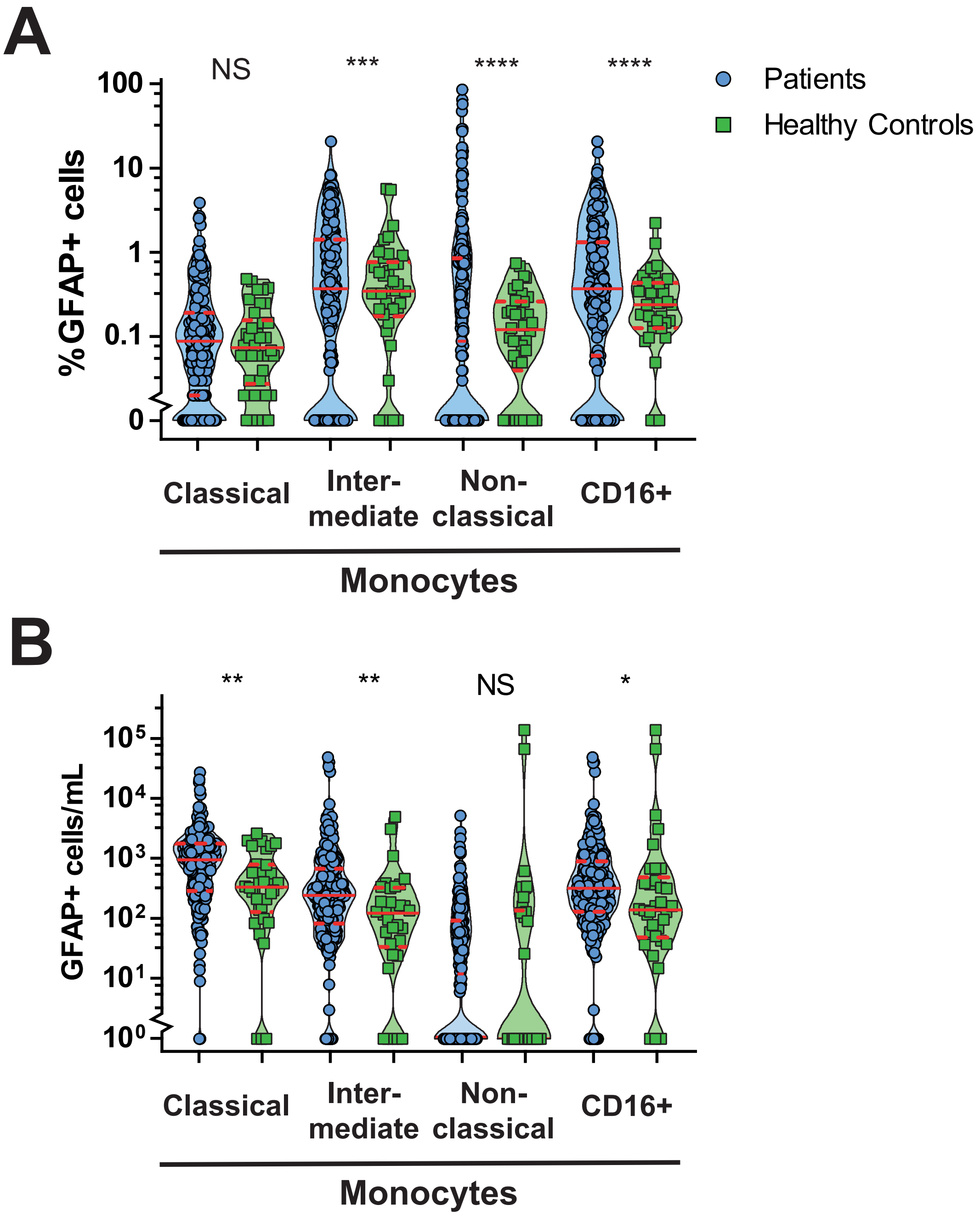

Supplementary Figure 3 Relative and absolute numbers of GFAP+ monocytes and their subsets in blood. (A) Violin plot with percentage of GFAP-positive cells per blood monocyte subset in glioma patients (blue circles)(N = 228) and healthy controls (green squares)(N = 38). **(B)** Violin plot of absolute number of GFAP+CD16+ monocytes per mL of blood, per monocyte subset in patients suspected of glioma (N = 228) vs healthy controls (N = 38). Red lines represent median and quartiles. Independent samples T-test; NS P > .05, *** P < .005, **** P < .001.

Supplementary Figure 4

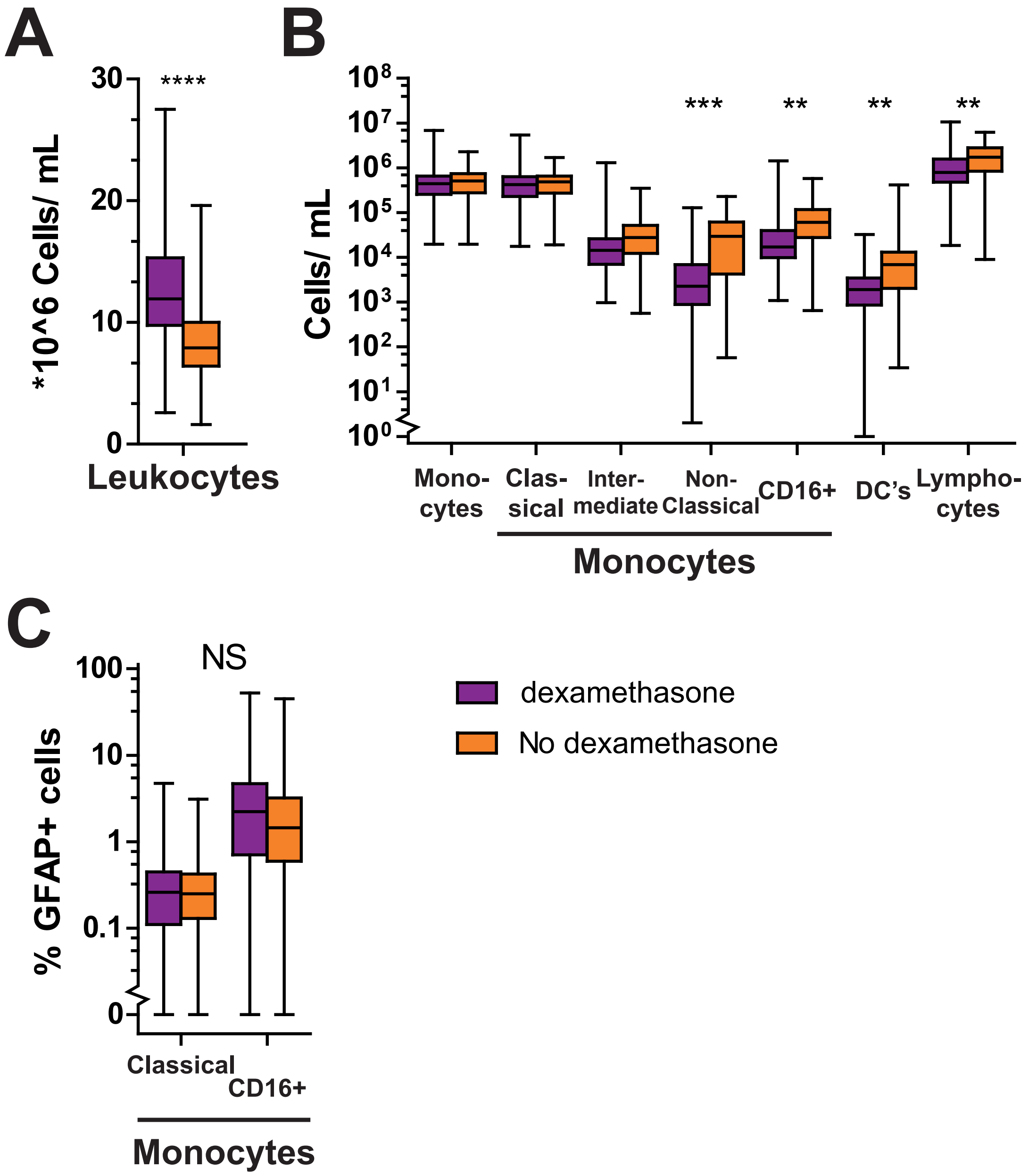

Supplementary Figure 4 Absolute monocyte and monocyte subset counts in blood of brain tumour patients and effect of prior dexamethasone therapy. (A) Absolute number of leukocytes per mL of blood in brain tumour patients with and without prior dexamethasone therapy. **(B)** Relative distribution of GFAP+ cells within classical and CD16+ monocytes in brain tumour patients according to prior usage or not of Dexamethasone. **(C)** Absolute number of cells per monocytic populations, dendritic cells (DC) and lymphocyte subset in blood according to the prior use of Dexamethasone therapy. **(B-C)** Boxes represent mean and 25th-75th percentile, whiskers show min-max. Student T-test; NS $P > .05$, ** $P < .01$, *** $P < .005$, **** $P < .001$.

Supplementary Figure 5

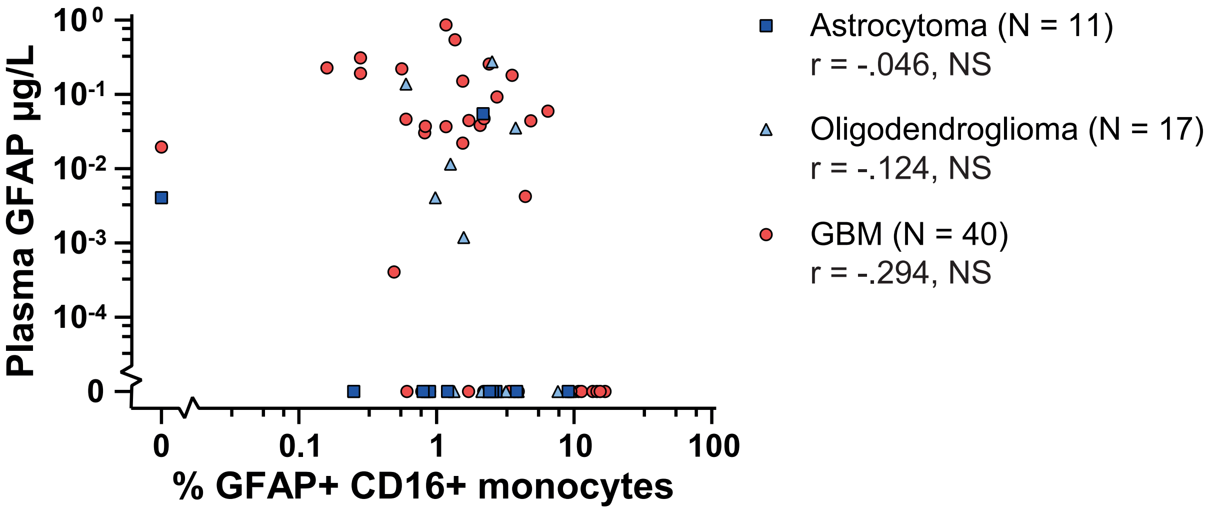

Supplementary Figure 5 %GFAP+CD16+ monocytes in glioma patients. Paired analysis of plasma GFAP (µg/L) and relative presence of GFAP+CD16 monocytes in blood of patients with astrocytoma (blue box), oligodendroglioma (light blue triangle) or GBM (red dot). The presented data are the mean of GFAP plasma analyses in duplicate. Pearson correlation: NS *P* > .05

Supplementary Figure 6

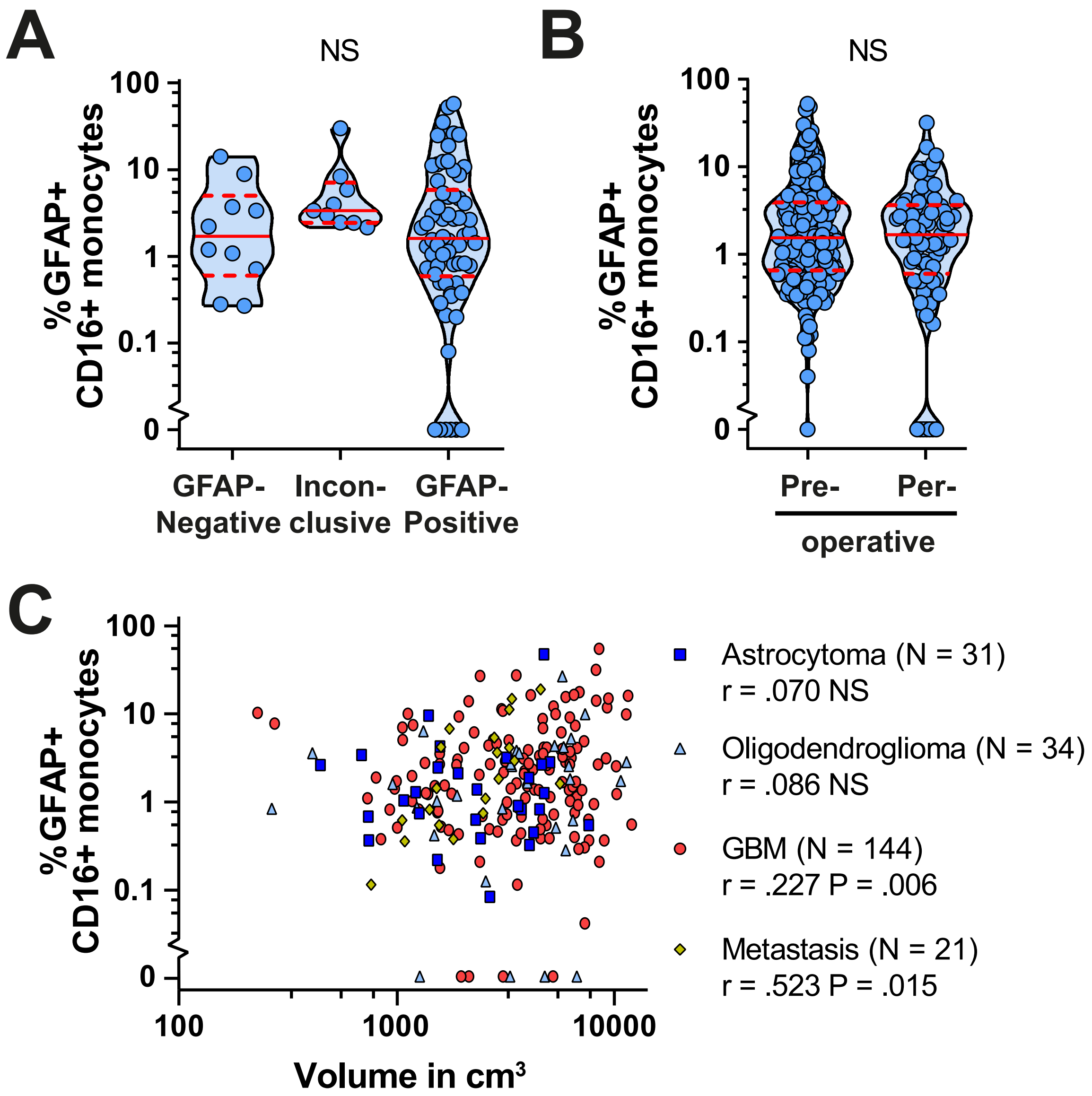

Supplementary Figure 6 %GFAP+CD16+ monocytes in blood and GFAP expression in the tumour, the time of sampling and tumour size. (A) %GFAP+CD16+ monocytes in GFAP- negative (N = 10), positive (N = 67) or inconclusive (N = 9) tumours. **(B)** %GFAP+CD16+ monocytes in blood of individual patients with first sampling at pre- vs per- operative time points (N = 187 and N = 72, respectively). **(C)** %GFAP+CD16+ monocytes and relationship with volume of the tumour per pathological grade. **(A-B)** Blue round circles represent individual patients and red lines represent mean ±SEM values. Kruskal Wallis **(A-B)** Pearson correlation (C); NS *P* > .05

Supplementary Figure 7

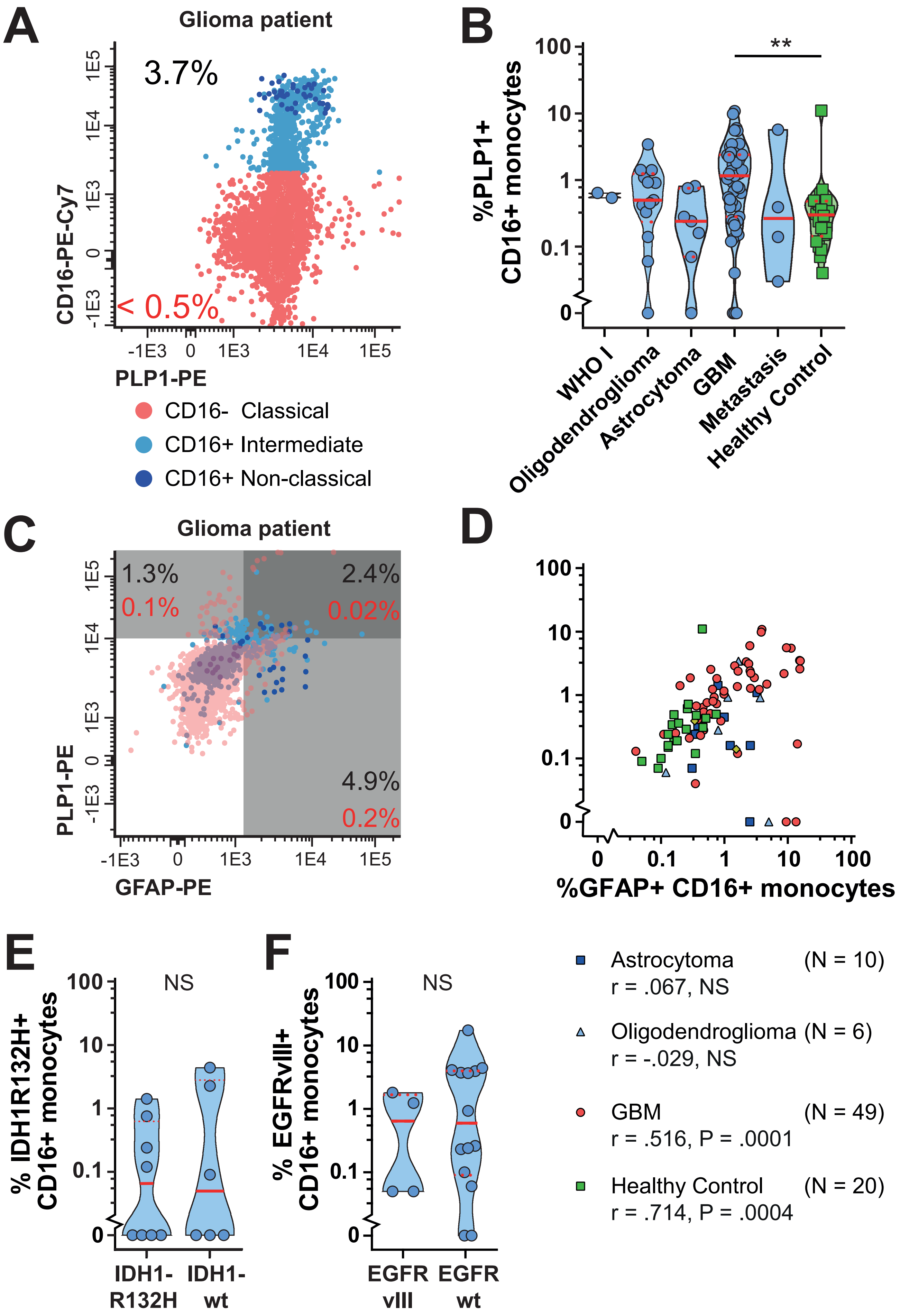

See next page for legend

Supplementary Figure 7 Tissue-/ disease- specific proteins other than GFAP evaluated for their presence in blood CD16+ monocytes. (A) Flow cytometric dotplot of PLP1-expression in monocyte subsets of a glioma-suspect patient. **(B)** Relative frequency of PLP1+CD16+ monocytes in metastasis and various grades of glioma. **(C)** Flow cytometric dotplot of intermediate and non-classical monocytes and their expression of GFAP and PLP1 in a glioma-suspect patient. **(D)** Dot plot of PLP1+ and GFAP+ expression in CD16+ monocytes in astrocytoma, oligodendroglioma, GBM and healthy controls. **(E)** Violin plot of relative frequency of IDH1R132H+CD16+ monocytes in blood of glioma patients with an IDH1R132H-mutated tumour (N = 8) vs IDH1-wild type (wt)(N = 6). **(F)** Relative frequency of EGFRvIII+CD16+ monocytes in blood of patients with an EGFRvIII mutated vs EGFR-wild type (wt). **(A-C)** Numbers represent the percentage in CD16+monocytes (black) and classical monocytes (red) carrying GFAP, PLP1 or GFAP and PLP1. **(B, E-F)** Red lines represent median and quartiles. Kruskal-Wallis **(B)**, Pearson correlation **(D)**, Students T-test **(E-F)**; NS $P > .05$, ** $P < .01$

Supplementary Figure 8

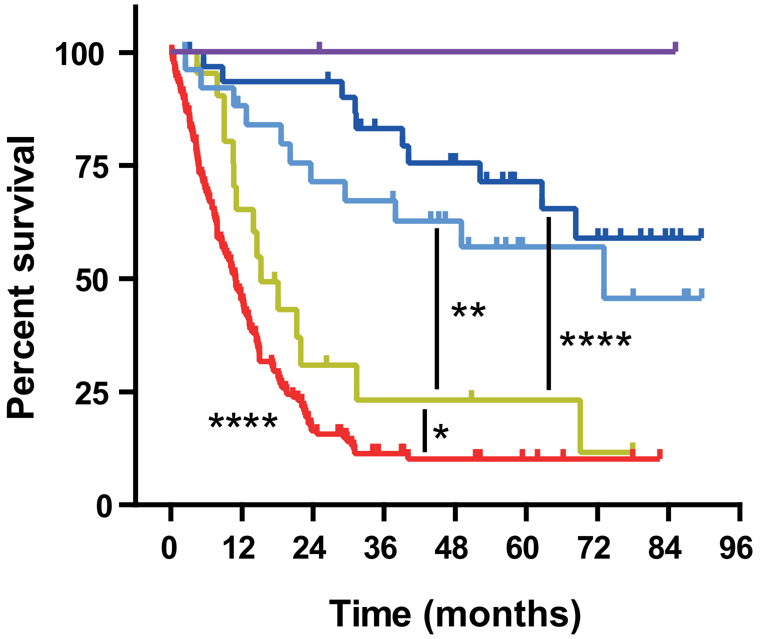

No. at risk										No. died	Median survival
WHO I	2	2	2	1	1	1	1	1	0	0	‡
Diffuse astrocytoma	28	21	17	16	11	5	5	3	0	13	73.1
Oligodendroglioma	32	28	28	22	19	12	9	3	0	11	>84.0
Glioblastoma	145	63	22	11	8	4	2	0	0	127	10.8
Metastasis	21	13	5	3	3	2	1	0	0	15	15.2

Supplementary Figure 8 OS of tumour patients per diagnostic group. Survival plot of various glioma grades and brain metastasis patients. OS of GBM patients was significantly decreased compared to all other (histopathological) grades apart from brain metastases. NS $P \geq .05$, * $P < .05$, ** $P < .01$, **** $P < .001$. ‡ No median calculated as numbers are too low and/or follow up is too short.

Supplementary Figure 9

A

Test		Tumor		
		Present	Absent	**Total**
	Positive	184	4	188
	Negative	44	22	66
	**Total**	228	26	

B

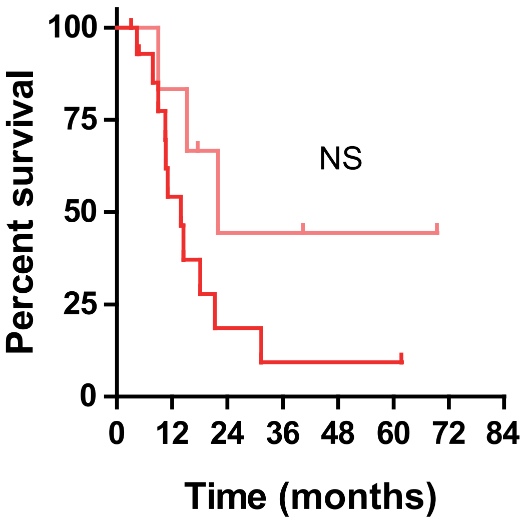

									**No. died**	**Median survival**
Meta <.75%	6	5	2	2	1	1	0	0	3	21.95
Meta >.75%	15	7	2	1	1	1	0	0	11	13.90

Supplementary Figure 9 Cross tabulation of test results and OS of brain metastasis patients. (A) Cross tabulation of the CD16+GFAP+ test results for each individual patient and healthy control. Positive test = ≥ 0.6% GFAP+CD16+monocytes. **(B)** OS of brain metastasis patients stratified for %GFAP+CD16+ monocytes. Survival curves of brain metastasis patients with GFAP+CD16+ monocytes below or over .75% of GFAP-positivity. Log-rank test, NS $P \geq .05$. Ticks mark censored cases.

Supplementary Table 1

Name	Conjugate	Clone	Producer
CD14	APC-H7	MO-P9	BD Bioscience, San José, CA
CD16	PE-Cy7	3G8	BD Bioscience, San José, CA
CD45	PacO	HI30	Invitrogen, Carlsbad, CA
HLA-DR	PCP-Cy5.5	L243	BD Bioscience, San José, CA
CD300e	APC	UP-H2	ImmunoStep, Salamanca, Spain
EGFRvIII	FITC	polyclonal	Biorbyt, Cambridge, UK
GFAP	PE	polyclonal	SantaCruz Biotechnology, Carlsbad, TX
IDH1R132H	FITC	DIAH09	Dianova, Hamburg, Germany
PLP1	FITC	J1/06	ImmunoStep, Salamanca, Spain

Supplementary Table 1 TiMaScan antibody panel, used for the flow cytometric studies with their commercial sources. APC, allophycocyanin; H7, hilite 7, Cy, cyanine; PacO, pacific orange; PCP, peridinin chlorophyll protein; PE, phycoerythrin. The basic set of TiMaScan antibodies (CD14, CD16, CD45, CD300e and HLA-DR) has become available from Cytognos, Salamanca, Spain.
